# Impact of diagnostic delay to the clinical presentation and associated factors in pediatric inflammatory bowel disease: a retrospective study

**DOI:** 10.1186/s12876-021-01938-8

**Published:** 2021-10-07

**Authors:** Emmiina Sulkanen, Marleena Repo, Heini Huhtala, Pauliina Hiltunen, Kalle Kurppa

**Affiliations:** 1 Tampere Center for Child Health Research, Tampere University, Arvo Ylpön katu 34, 33520 Tampere, Finland; 2 Department of Pediatrics, Tampere University Hospital, Tampere, Finland; 3 Faculty of Social Sciences, Tampere University, Tampere, Finland; 4 Department of Pediatrics, Seinäjoki University Hospital, Seinäjoki, Finland; 5 University Consortium of Seinäjoki, Seinäjoki, Finland

**Keywords:** Pediatric inflammatory bowel disease, Crohn’s disease, Ulcerative colitis, Diagnostic delay

## Abstract

**Background:**

Undelayed diagnosis is thought to be a major determinant for good prognosis in pediatric inflammatory bowel disease (PIBD). However, factors predicting diagnostic delay and the consequences of this remain poorly defined. We investigated these issues in a well-defined cohort of PIBD patients.

**Methods:**

Comprehensive electronic data were collected from 136 PIBD patients retrospectively. Diagnostic delay was further classified into < 6 and ≥ 6 months, and < 12 and ≥ 12 months. Logistic regression was used to calculate whether the delay was associated with clinical features and/or risk of complications and co-morbidities at diagnosis.

**Results:**

The median age of patients was 12.4 years and 43.4% were females. Altogether 35.5% had Crohn´s disease (CD), 59.1% ulcerative colitis (UC) and 6.6% IBD undefined (IBD-U). The median delay before diagnosis was 5.0 months in all, 6.6 months in CD, 4.1 months in UC, and 9.8 months in IBD-U (UC vs. CD, *p* = 0.010). In all but IBD-U most of the delay occurred before tertiary center referral. Abdominal pain predicted a delay > 6 months in all PIBD (OR 2.07, 95% CI 1.00–4.31) and in UC patients (3.15, 1.14–8.7), while bloody stools predicted a shorter delay in all PIBD (0.28, 0.14–0.59) patients and in CD (0.10, 0.03–0.41) patients. A delay > 6 months was associated with a higher frequency of complications (2.28, 1.01–5.19).

**Conclusions:**

Delay occurred mostly before specialist consultation, was longer in children presenting with abdominal pain and in CD and was associated with risk of complications. These findings emphasize the roles of active case-finding and prompt diagnostic evaluations.

**Supplementary Information:**

The online version contains supplementary material available at 10.1186/s12876-021-01938-8.

## Background

Up to 25% of inflammatory bowel disease (IBD) patients are diagnosed in childhood, and while ulcerative colitis (UC) is the most common form of IBD in adults*,* the distribution of pediatric subtypes varies markedly by age and region [1–5]. Children often have extensive intestinal involvement and aggressive disease behavior [1, 5] which, together with the risk of permanent complications in growing children, emphasizes the importance of promptly initiated effective treatment. This conclusion is further supported by evidence that diagnostic delay increases the risk for complicated disease course in IBD patients diagnosed in adulthood [6, 7].

At present, however, studies on the median length and factors associated with diagnostic delay in children with IBD remain scant. In addition, whether the delay affects initial disease severity and risk of complications and co-morbidities in pediatric IBD (PIBD) patients remains debatable. For example, while Ricciuto et al. reported delay to be associated with risk of complicated disease course [8], in a recent meta-analysis this was observed only in adult IBD patients [9]. A better understanding of the consequences of diagnostic delay in PIBD is even more important in the era of modern biological therapies, which—although often effective—are highly immunosuppressive and limited in number and should thus be optimally targeted.

Finland has a high prevalence if PIBD [10, 11] and new diagnostic and follow-up tools have been actively applied in clinical practice [12–14]. With the help of these benefits and due to availability of systemically maintained patient records, we aimed to investigate the effect of a diagnostic delay to the clinical presentation and associated factors in PIBD.

## Materials and methods

### Patients and study design

The observational retrospective study was conducted at the Center for Child Health Research, Tampere University and at the Department of Pediatrics, Tampere University Hospital. The hospital is a tertiary center for a population of approximately 150,000 children and the main referral site for suspected IBD patients. The study cohort was formed by first collecting comprehensive clinical and laboratory data from electronic medical records of all consecutive children (age < 18 years) undergoing upper and/or lower gastrointestinal endoscopies and other diagnostic investigations between January 2007 and October 2014 in the Pediatric Gastroenterology Unit. The search resulted in altogether 2395 endoscopies conducted on 1263 children. Next, all patients with a confirmed IBD diagnosis in childhood were selected for the study analyses as further described below, thus the IBD diagnosis represented the index date.

The study design and the collection of the medical data were approved by the Department of Pediatrics, Tampere University Hospital. All data was analyzed anonymously and the Ethical Guidelines of the Declaration of Helsinki were strictly followed. Under the national regulations, no approval of the Ethics Committee was needed for this registry-based study.

### Clinical and laboratory data

The clinical data collected included demographic and anthropometric information on the duration and nature of the disease-associated symptoms before the PIBD diagnosis, presence of IBD in relatives, and presence and nature of possible associated complications and/or co-morbidities.

The symptoms were further classified into abdominal pain, blood in stool, diarrhea, poor growth, constipation, nausea/vomiting and other (e.g., anorexia, delayed puberty, tiredness, recurrent oral symptoms, arthritis, fever, and ocular manifestations). Poor growth was defined by the physician based on the age- and sex-adjusted Finnish reference standards [15, 16].

Complications were further categorized into perianal disease (fistulas, fissures, skin tags), structural or surgical complications (abscesses other than perianal fistulas, intestinal strictures/stenoses, and disease-related gastrointestinal surgery). Possible co-morbidities included autoimmune hepatitis, primary sclerosing cholangitis, uveitis, pancreatitis, ankylosing spondylitis, peripheral arthritis, pyoderma gangrenosum, venous thromboembolism, and gastrointestinal malignancy.

The laboratory parameters collected comprised blood hemoglobin (Hb, reference values from 95–150 to 130–230 g/l depending on age and sex) [17], C-reactive protein (CRP, < 10 mg/l), erythrocyte sedimentation rate (ESR, < 15 mm/h), plasma albumin (Alb, from 35–46 to 37–51 g/l) and fecal calprotectin (< 100 µg/g [18]).

The presence and nature of symptoms were collected before the index date and laboratory parameters were taken either at the time of endoscopic investigations or shortly before. The recorded complications included those found during the initial diagnostic investigations (endoscopies, imaging studies etc.) carried out either at the time of IBD diagnosis or during the few following months.

### Diagnostic investigations and disease classifications

Standardized sampling protocol during the endoscopies was conducted throughout the study period, the routine practice including obtaining ≥ 2 representative mucosal forceps biopsies from the esophagus, gastric body, antrum and duodenum during esophagogastroduodenoscopy (EGD), and from the rectum, sigmoid/descending colon, ascending colon, cecum, and terminal ileum during ileocolonoscopy [17]. Additional biopsies were taken based on the clinical scenario and endoscopic findings. The mucosal specimens were evaluated systemically by pathologists specialized in the pediatric alimentary tract. Variable imaging studies were also conducted to establish the subtype and extent of IBD, including X-ray studies and computed tomography, ultrasonography, magnetic resonance imaging, and capsule endoscopy.

The IBD subtype, as defined by the physician in charge on the basis of the aforementioned investigations and international guidelines [19, 20], was recorded for each patient. Disease location was moreover verified and further classified into ileal, colonic, ileocolonic, and as proximal or distal to the ligament of Treitz (CD) or into rectal, left sided, and extensive/pancolitis (UC) [4, 21].

### Definitions

Diagnostic delay was defined as time from first relevant symptoms to IBD diagnosis and reported in months. The delay was further subdivided into time from symptom onset to first healthcare visit and from subsequent referral to eventual IBD diagnosis. The former was based on patient/parental recall and the latter on exact dates collected from the patient records. The delay was further classified into < 6 and ≥ 6 months (including also > 12 months), and < 12 (including also < 6 months) and ≥ 12 months. The delay between < 6 and ≥ 6 months was the main outcome for the association analyses.

### Statistics

The patient characteristics are presented as number of cases and/or percentage distributions. Most of the quantitative variables were not normally distributed when assessed by the Shapiro–Wilk method and were thus, for the sake of simplicity, all expressed as medians with lower and upper quartiles. Chi-square test, Fisher’s exact test or Kruskal–Wallis test were used in the statistical analyses as appropriate, considering *P* value < 0.05 significant. Associations between patient characteristics and diagnostic delay of ≥ 6 and ≥ 12 months were calculated for all PIBD patients and separately for those with CD and UC using binary logistic regression where the delay of < 6 months represented the reference. The results were given as odds ratios (OR) with 95% confidence intervals. All statistical analyses were performed using IBM SPSS Statistics for Windows, Version 26.0 (IBM Corp, Armonk, NY, USA).

## Results

Altogether 139 children received an IBD diagnosis during the study period, but three were excluded because of insufficient data on the delay. Of the remaining 136 children, 48 (35.3%) had CD, 79 (58.1%) UC, and nine (6.6%) IBD unclassified (IBD-U) (Table 1). In general, children with IBD-U were younger, less often females and more often hypoalbuminemic at diagnosis than CD and UC patients, whereas those with CD had less often anemia, bloody stools, and increased calprotectin but more often constipation and poor growth than the two other subgroups (Table 1). Altogether 131 (96.3%) of the patients had undergone both colonoscopy and EGD and four (2.9%) colonoscopy only.

**Table 1:** Baseline characteristics of 136 children with IBD, further divided into CD, UC and IBDU

	All IBD n = 136	CD n = 48	UC n = 79	IBDU n = 9
	%	%	%	%
Demographic data				
Age, median (range), year	12.4 (1.3, 16.6)	12.2 (1.5, 16.6)	12.7 (1.3, 16.4)	9.4 (3.4, 15.2)
Females	43.4	45.8	40.5	55.6
IBD in relatives	17.6	14.6	22.2	17.6
Laboratory values				
Fecal calprotectin > 100 μg/g	88.2	79.4	92.5	100
Elevated^1^ ESR and/or CRP	64.9	63.0	64.5	77.8
Anemia	57.1	44.7	63.6	57.1
Hypoalbuminemia	43.7^2^	40.7^3^	42.3^4^	62.5^5^
Symptoms				
Abdominal pain	65.4	70.8	62.0	66.7
Blood in stool	62.5	37.5	77.2	66.7
Diarrhea	52.2	43.8	59.5	33.3
Poor growth	34.6	47.9	29.1	11.1
Constipation	9.6	14.6	7.6	0
Nausea or vomiting	8.8	10.4	7.6	11.1
Other^6^	55.1	58.3	54.4	44.4

IBD, inflammatory bowel disease; CD, Crohn’s disease; UC, ulcerative colitis; IBDU, inflammatory bowel disease undefined; ESR, erythrocyte sedimentation rate; CRP, C-reactive protein. ^1^ESR > 15 mm/h or CRP > 10 mg. Data was available from all patients in demographic data and symptoms, and in laboratory values from > 80% except in ^2^38, ^3^11, ^4^22 and ^5^5. ^6^E.g. tiredness, fever, oral symptoms, arthralgia

The median diagnostic delay in PIBD was 5.0 (quartiles 2.5, 9.3) months. In more detailed analysis, the longest median delay was seen in patients with IBD-U and the shortest in those with UC, but there was a wide variation in individual delays and the difference was statistically significant only between CD and UC (Table 2). Of note, in both CD and UC most of the delay occurred before referral to hospital, while the opposite was true in IBD-U (Table 2).

**Table 2:** Diagnostic delay before referral and before diagnosis in 136 children with inflammatory bowel disease (IBD), further divided into Crohn’s disease (CD), ulcerative colitis (UC), and IBD unclassified (IBDU)

Delay	All IBD (n = 136)	CD (n = 48)	UC (n = 79)	IBDU (n = 9)
Before referral				
Median (quartiles), months	3.3 (1.0, 7.0)	5.0 (2.3, 11.5)	3.0 (1.0, 6.0)	2.0 (2.0, 13.0)
≥ 6 months, %	37.5	47.7	30.7	44.4
≥ 12 months, %	18.0	25.0	13.3	22.2
Before diagnosis				
Median (quartiles), months	5.0 (2.5, 9.3)	6.6 (3.5, 12.6)^1^	4.1 (2.0, 8.0)	9.8 (2.6, 21.0)
≥ 6 months, %	45.6	54.2	39.2	55.6
≥ 12 months, %	19.9	27.1	13.9	33.3

^1^*P* = 0.01 compared with UC. There were no other statistically significant differences between the groups

The anatomical distribution of CD and UC, as defined endoscopically and/or based on imaging findings, is presented in Additional file 1: Figure S1. Neither median length of delay nor percentage of delay exceeding 6/12 months was significantly associated with disease location at diagnosis (data not shown).

Of the patient characteristics studied, presence of abdominal pain was significantly associated with diagnostic delay ≥ 6 months when analyzed in all patients, whereas bloody stools increased the likelihood of a delay < 12 months (Table 3). In a stratified analysis, abdominal pain was significantly associated with a delay ≥ 6 months in UC patients and bloody stools with a delay < 6 months in CD patients (Additional file 2: Table S1).

**Table 3:** Associations between the patient characteristics and diagnostic delay in 136 children with inflammatory bowel disease

	Delay ≥ 6 months			Delay ≥ 12 months		
	%	OR^1^	95% CI	%	OR^1^	95% CI
Demographic data						
Male (vs. female)	59.7	1.26	0.64–2.49	63.0	1.39	0.58–3.31
Age 13–17 year vs. < 13 year	37.1	0.73	0.37–1.46	40.7	0.98	0.42–2.30
IBD in relatives	14.5	0.67	0.27–1.65	18.5	1.08	0.36–3.20
Symptoms						
Abdominal pain	74.2	**2.07**	**1.00**–**4.31**	74.1	1.66	0.64–4.26
Diarrhea	51.6	0.96	0.49–1.88	48.1	0.82	0.35–1.90
Blood in stool	46.8	**0.28**	**0.14**–**0.59**	40.7	**0.33**	**0.14**–**0.77**
Poor growth	32.2	0.83	0.41–1.69	37.0	1.15	0.48–2.75
Constipation	11.3	1.44	0.46–4.54	14.8	1.93	0.55–6.83
Nausea or vomiting	11.3	1.76	0.53–5.84	7.4	1.39	0.35–5.52
Other^2^	59.7	1.40	0.71–2.77	63.0	1.50	0.63–3.56

Bolded values are statistically significant

*CI* confidence interval, *OR* odds ratio. ^1^Binary logistic regression analysis; ^2^E.g. tiredness, fever, oral symptoms, arthralgia

Altogether, 22.8% of all 136 patients had one or more complication and 8.8% a co-morbidity, the most common of these being perianal disease (Table 4). Complications and co-morbidities were significantly less likely in children with diagnostic delay of < 6 months than in those with longer delay when evaluated as a whole, but not—despite a similar trend in each three categories—in separate analysis (Table 4).

**Table 4:** Association between the length of diagnostic delay and presence of complications and co-morbidities in 136 children with inflammatory bowel disease

	%	OR	95% CI
Total			
< 6.0 months	16.2	1	
≥ 6.0 months	30.6	2.28	**1.01**–**5.19**
≥ 12.0 months	37.0	2.47	0.99–6.15
Perianal disease			
< 6.0 months	8.1	1	
≥ 6.0 months	24.2	1.93	0.65–5.75
≥ 12.0 months	18.5	2.25	0.70–7.24
Abscesses, fistulas, strictures, surgery			
< 6.0 months	2.7	1	
≥ 6.0 months	8.1	3.16	0.59–16.90
≥ 12.0 months	11.1	3.28	0.69–15.60
Co-morbidity^1^			
< 6.0 months	5.4	1	
≥ 6.0 months	12.9	2.59	0.74–9.06
≥ 12.0 months	11.1	1.35	0.35–5.52

Bolded values are statistically significant

^1^Autoimmune hepatitis, primary sclerosing cholangitis, uveitis, pancreatitis, venous thromboembolism, ankylosing spondylitis, peripheral arthritis, pyoderma gangrenosum, gastrointestinal malignancy. *OR* odds ratio, *CI* confidence interval

## Discussion

We found the median diagnostic delay in all PIBD patients to be 5.0 months. Moreover, the delay was significantly longer in children with CD than in those with UC. Both of these findings are in line with those of the earlier pediatric studies by Buderus et al., Schoepfer et al., Sawczenko et al. and Ricciuto et al. [23–25]. In addition, Timmer et al. [26] reported a median diagnostic delay of 4.0 months, Arcos-Machancoses et al. [27] of 2.8 months and Ricciuto et al. [8] of 4.2 months when considering PIBD as a whole, while Schoepfer et al. [9] observed a delay of 3.0 months in CD. Interestingly, in some older studies in particular, the delays have been somewhat longer, probably reflecting the ongoing improvements in the clinical case finding and diagnostic tools of PIBD [28, 29], although differences in the study design and methodology might also have affected. However, suggesting significant country-related differences, delay has also been quite long in some recent studies [30].

In more detailed analysis, the time between symptom onset and referral to a specialist was the main cause of delay in both CD and UC. The few existing reports on this issue have been somewhat inconsistent [23–25, 27, 30]. In line with us, Ricciutto et al. and Mouzan et al. reported time before referral to account for most part of the delay in PIBD [25, 30]. In contrast, Schoepfer et al. found the duration from referral to diagnosis to be longer than that before the first healthcare contact [23]. Of note, we found children with IBD-U to differ from those with CD and UC, as the former had a longer median delay which also occurred mostly after referral. This is logical given the ambiguous definition of IBD-U, which was also our reason for not including this subgroup in the main analyses. Indeed, variable diagnostic definitions and inclusion criteria could contribute to the reported differences between studies [8, 23–25].

Of the factors associated with delay, bloody stools predicted a short delay in all PIBD patients and also separately in CD, while abdominal pain was associated with long delay in all and in those with UC. Bleeding/bloody stools were also associated with shorter delay in PIBD in the studies by Ricciuto et al. [25] and Sawczenko el al. [24]. Moreover, Ricciuto et al. reported diarrhea to be associated with shorter delay and Spray et al. [28] symptoms other than diarrhea with longer delay. Other factors reportedly associated with longer delay are particularly young age [24, 26, 27, 30] and isolated small-bowel disease/ileal location in CD [25, 26, 29, 30]. These findings are consistent with the higher risk for long delay in CD, as it often presents with vague symptoms that can be difficult for parents and physicians to recognize. The inconsistent results between studies are likely caused by differences in study populations and definitions of clinical features. For example, we had only a few cases presenting with fever or isolated small bowel disease.

Emphasizing the importance of undelayed diagnosis, here the delay was associated with increased risk for complications and co-morbidities. Although significant only when counted as a whole, there was a similar trend in each subcategory. Earlier results have again been inconsistent. Ricciutto et al. reported delay to be associated with increased risk for intestinal strictures and fistulas [8], whereas Schoepfer et al. [9] found longer delay to predict *lower* risk for these complications and for intestinal surgery, the latter also being reported by Krishna et al. [31]. These seemingly contradictory findings may be due to the fact that severe presentation may both predict increased risk for complications [32] and also hasten the diagnostic evaluations. It is evident that the direction of this association is determined by many factors, including age at diagnosis, disease location, and total delay duration. Of note, several earlier studies considering poor growth as a complication rather than as a symptom have reported it to be associated with delay [24–26, 28, 29]. This is again in line with the elevated delay risk in CD.

### Strengths and limitations of the study

Our main strength was the well-defined cohort of consecutive PIBD patients. Specifically, the use of systemically maintained patient records afforded us accurate clinical and histological data. The retrospective design was an unavoidable weakness that hampered precise assessment of the delay. However, in most cases this information was already recorded at the time of the initial evaluations, and thus presumably accurate regarding the main outcomes. Additionally, recall bias could have had affected our findings of the delay before referral to the tertiary centre. It must also be realized that a part of the delay could be an in-time delay since some time is usually required to make the diagnostic investigations. The fact that we only included children diagnosed in one tertiary center may impair the generalizability of the results but, on the other hand, our hospital is the main referral site for all cases with clinical suspicion of PIBD in an area of approximately 150,000 children. Besides, a single-center design can be expected to provide more uniform diagnostic procedures and patient registers. A clear limitation was the unsystematic use of laboratory parameters and lack of long-term follow-up data. Nor did we include mode of treatment or long-term follow-up data in our analyses, as the former may reflect more the general severity of the disease, and there has been a major paradigm shift in the treatment of IBD during the study period [33]. Finally, the nowadays widely used IBD activity scores were not systemically used during the whole study period and thus could not be included in the study analyses.

## Conclusions

To conclude, we found diagnostic delay in PIBD to be longer in children with CD and to occur mostly before the specialist consultation. Furthermore, there was an association between longer delay and increased risk for complications and co-morbidities. These findings emphasize the role of active case-finding and low-threshold use of modern non-invasive diagnostic markers in primary care, as well as rapid consultation in case of PIBD suspicion. Aiming to reduce delay to a minimum is even more important in this era of modern biological drugs and aggressive top-down therapy.

## Supplementary Information

**Additional file 1: Figure S1**. Location of the disease at diagnosis in the 127 study children with either Crohn’s disease (CD) or ulcerative colitis (UC).

**Additional file 2: Supplementary Table 1.** Disease location in 76 children with ulcerative colitis (UC) and 47 children with Crohn’s disease (CD) with and without histologic upper gastrointestinal (UGI) findings.

## Data Availability

Due to protect patient privacy, the original data used to support the findings of this study cannot be shared.

## Abbreviations

PIBD: Pediatric inflammatory bowel disease
CD: Crohn’s disease
UC: Ulcerative colitis
IBD-U: IBD undefined
IBD: Inflammatory bowel disease
EGD: Esophagogastroduodenoscopy
