# Supplementary figures and images for: Impact of diagnostic delay to the clinical presentation and associated factors in pediatric inflammatory bowel disease: a retrospective study
Source: BMC Gastroenterol. 2021 Oct 7;21:364. doi: 10.1186/s12876-021-01938-8 (PMC8495911; doi:10.1186/s12876-021-01938-8)

## Slide 1
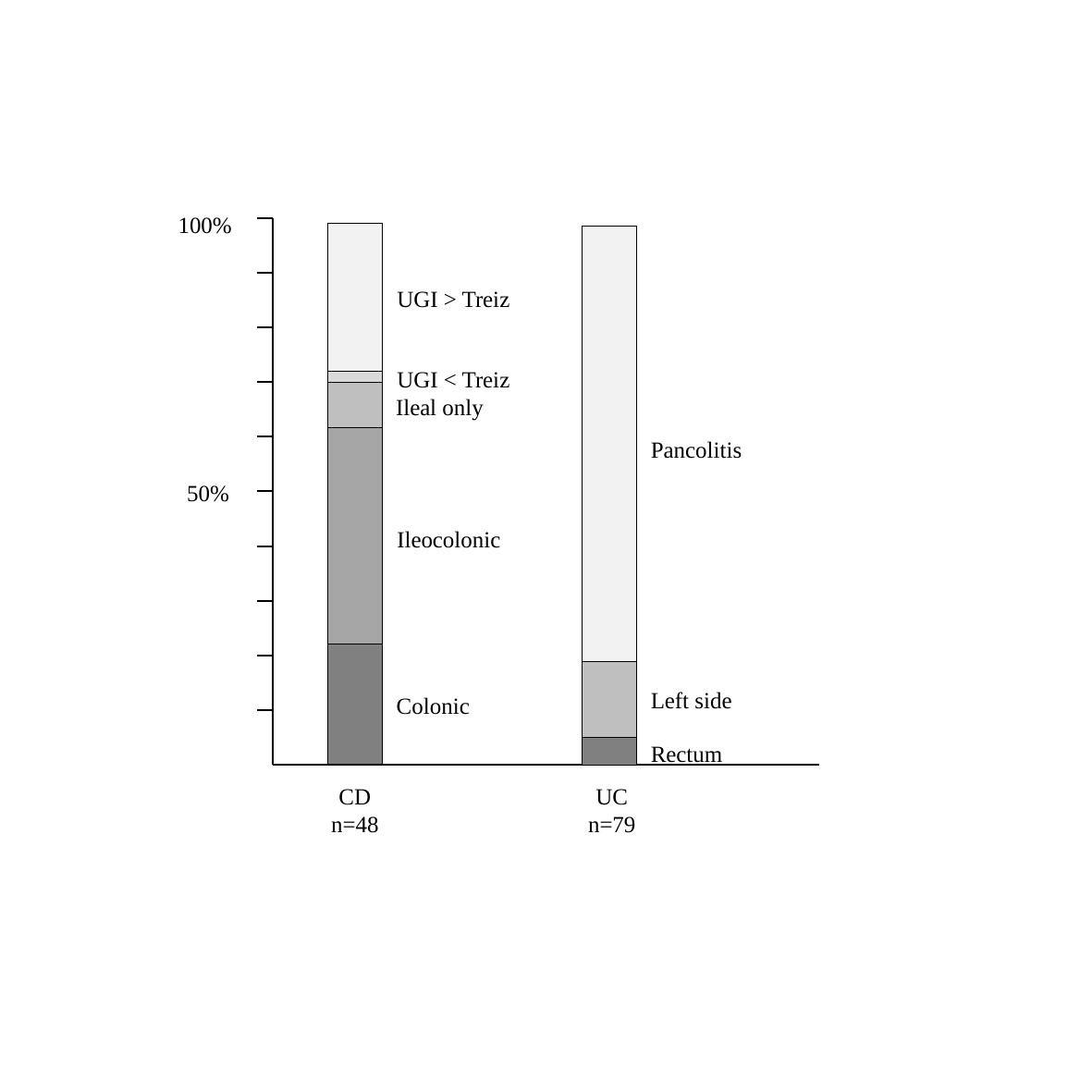

100%
UGI > Treiz
UGI < Treiz
Ileal only
Pancolitis
50%
Ileocolonic
Left side
Colonic
Rectum
CD n=48
UC n=79

Supplement: Supplementary file 1 — Additional file 1: Figure S1. Location of the disease at diagnosis in the 127 study children with either Crohn’s disease (CD) or ulcerative colitis (UC). [file 12876_2021_1938_MOESM1_ESM.pptx]
