# Supplementary material for: Impact of diagnostic delay to the clinical presentation and associated factors in pediatric inflammatory bowel disease: a retrospective study
Source: BMC Gastroenterol. 2021 Oct 7;21:364. doi: 10.1186/s12876-021-01938-8 (PMC8495911; doi:10.1186/s12876-021-01938-8)
Supplement: Supplementary file 2 — Additional file 2: Supplementary Table 1. Disease location in 76 children with ulcerative colitis (UC) and 47 children with Crohn’s disease (CD) with and without histologic upper gastrointestinal (UGI) findings. [file 12876_2021_1938_MOESM2_ESM.docx]

| **Supplementary Table 1.** Associations between patient characteristics and diagnostic delay of >6 months in pediatric CD or UC | | | | | | | | | | | |
| --- | --- | --- | --- | --- | --- | --- | --- | --- | --- | --- | --- |
|  | |  | CD, n=48 | | |  |  | UC, n=79 | | |  |
|  | | % | | OR^1^ | 95% CI | | % | | OR^1^ | 95% CI | |
| *Demographic data* | |  | |  |  | |  | |  |  | |
| Male (*vs.* female) | 65.4 | | 2.73 | 0.85-8.81 | | 54.8 | | 0.73 | 0.29-1.82 | |  |
| Age 13-17 yr *vs.* <13 yr | 38.5 | | 1.09 | 0.34-3.54 | | 41.9 | | 0.72 | 0.29-1.80 | |  |
| IBD in relatives | 7.7 | | 0.28 | 0.05-1.64 | | 16.1 | | 0.73 | 0.22-2.39 | |  |
|  | |  | |  |  | |  | |  |  | |
| *Symptoms* | |  | |  |  | |  | |  |  | |
| Abdominal pain | 73.1 | | 1.27 | 0.36-4.41 | | 77.4 | | 3.15 | 1.14-8.70 | |  |
| Diarrhea | 50.0 | | 1.75 | 0.55-5.58 | | 58.1 | | 0.91 | 0.36-2.27 | |  |
| Blood in stool | 15.4 | | 0.10 | 0.03-0.41 | | 71.0 | | 0.56 | 0.20-1.63 | |  |
| Poor growth | 46.2 | | 0.86 | 0.28-2.67 | | 25.8 | | 0.77 | 0.28-2.10 | |  |
| Constipation | 15.4 | | 1.15 | 0.23-5.81 | | 9.7 | | 1.61 | 0.30-8.52 | |  |
| Nausea or vomiting | 11.5 | | 1.30 | 0.20-8.61 | | 12.9 | | 3.41 | 0.59-19.9 | |  |
| Other^2^ | 65.4 | | 1.89 | 0.59-6.04 | | 58.1 | | 1.27 | 0.51-3.17 | |  |
| CI, Confidence interval; CD, Crohn’s disease; UC, ulcerative colitis; OR, odds ratio. ^1^Binary logistic regression analysis; ^2^E.g. tiredness, fever, oral symptoms, arthralgia | | | | | | | | | | | |
